# Development of long-term event memory in preverbal infants: an eye-tracking study

**DOI:** 10.1038/srep44086

**Published:** 2017-03-08

**Authors:** Tamami Nakano, Shigeru Kitazawa

**Affiliations:** 1 Graduate School of Frontier Biosciences, Osaka University, Osaka 5650871, Japan; 2 Graduate School of Medicine, Osaka University, Osaka 5650871, Japan; 3 Precursory Research for Embryonic Science and Technology (PREST), Japan Science and Technology (JST), Tokyo 1020076, Japan

## Abstract

The development of long-term event memory in preverbal infants remains elusive. To address this issue, we applied an eye-tracking method that successfully revealed in great apes that they have long-term memory of single events. Six-, 12-, 18- and 24-month-old infants watched a video story in which an aggressive ape-looking character came out from one of two identical doors. While viewing the same video again 24 hours later, 18- and 24-month-old infants anticipatorily looked at the door where the character would show up before it actually came out, but 6- and 12-month-old infants did not. Next, 12-, 18- and 24-month-old infants watched a different video story, in which a human grabbed one of two objects to hit back at the character. In their second viewing after a 24-hour delay, 18- and 24-month-old infants increased viewing time on the objects before the character grabbed one. In this viewing, 24-month-old infants preferentially looked at the object that the human had used, but 18-month-old infants did not show such preference. Our results show that infants at 18 months of age have developed long-term event memory, an ability to encode and retrieve a one-time event and this ability is elaborated thereafter.

If we lose our memory of personal events and experiences, we might become confused regarding who we are, where we are and what we should do. Tulving first coined the term ‘episodic memory’ to describe this autobiographical event memory and differentiate it from other forms of declarative memory^1^. This ability of episodic memory becomes apparent around the age of 3–4 years^2^,^3^,^4^. However, the developmental precursors of episodic memory in preverbal infants remain unclear because this type of memory is difficult to assess in the absence of language^5^.

To examine event-based long-term memory in preverbal infants, Russel and Thompson^6^ utilized an ‘episodic-like’ memory paradigm, which was originally used to demonstrate memory in scrub jays as to *where* and *when* particular food items (*what*) were cached^7^. In their study, one experimenter placed a toy in each of two boxes and then another experimenter removed one toy from one of the boxes in front of infants. After a delay of 24 hours, the infants were urged to find the toy and those infants over 21 months of age reached for the correct box, in which the toy remained. These results suggest that infants around their second birthday have an ability to remember two different events (placement and removal) that occurred at different locations (boxes).

In contrast to the reported late emergence in the development of long-term event memory, other researchers have reported earlier emergence of other types of long-term memory. Using a visual paired-comparison task, Pascalis *et al*.^8^ reported that 3- and 6-month-old infants remembered human faces after a delay of 24 hours. Richmond *et al*.^9^ employed a habituation task to show that 9-month-old infants remembered spatial arrangements of objects over a 24-hour delay. In addition, a deferred imitation paradigm and a mobile conjugate reinforcement paradigm revealed that 6-month-old infants were able to reproduce a particular action after delays of 24 hours^10^,^11^ and two weeks^12^, respectively. Because the performance on all of these tasks in the test phase after a delay was influenced by context changes in the encoding phase, the memory system involved in these tasks is considered declarative^5^. However, during the encoding phase of these tasks, an identical stimuli/action was repeatedly presented^8^,^9^,^10^, or infants were trained to perform a particular action several times^12^. Therefore, these studies did not necessarily examine an ability of long-term memory of one-time events in preverbal infants.

Kano and Hirata^13^ recently invented a novel eye-tracking method to investigate long-term memory of one-time events in non-verbal great apes. In the method, identical movies were presented to the great apes twice, with an interval of 24 hours and their eye movements were compared across the two presentations. The results revealed that great apes make anticipatory looks at locations or objects related to future events based on the long-term memory of a previous single experience. Because infants around 6 months of age have already exhibited anticipatory looking behaviour^14^,^15^, this paradigm is appropriate for examining an ability of long-term event memory not only in non-verbal animals but also in preverbal human infants. The present study, therefore, utilized the same video stimuli and paradigm as that in the great apes study for a wide age range of preverbal infants to investigate the developmental process of long-term memory of a one-time event. In the previous great apes study, researchers created two half-minute movie clips in which the critical test events were contextualized within emotional events because emotion enhances long-term event memory^16^. Infants at the age of four months old are reported to form a kind of long-term memory for a stressful social events as revealed by measuring cortisol and autonomic responses^17^,^18^. Thus, we expected that emotional contexts in the movie would enhance long-term memory of a one-time event in preverbal infants at the age of 6 months old and above. The first clip was designed for testing long-term event memory related to location. In the present study, infants from 6 to 24 months of age viewed this video story once, in which an actor wearing a King Kong suit (KK) came out from one of two identical doors (the target door) and attacked a human actor. Using a gaze-tracking system, we examined whether the infants would anticipatorily look at the target door before the appearance of KK on their second viewing of the same movie, 24 hours later. The second clip was designed for testing the long-term event memory related to objects. Infants from 12 to 24 months of age viewed this second video clip once, in which a human actor beaten by KK grabbed one of two different objects (the target object) and hit back KK with it. On the second viewing of the same movie following a 24-hour delay, we examined whether the infants would increase viewing time of the objects, especially for the target object, before the actor reached for the object. To dissociate object memory from location memory, we had half of the infants for each age group view the same movie but with the object location switched on the second day.

## Methods

### Participants

In Experiment 1, participants consisted of 64 healthy, full-term infants divided into four groups based on age: sixteen 6-month-olds (6 M, mean age: 191 days, range: 180–204 days; 7 females), sixteen 12-month-olds (12 M, mean age: 377 days, range: 365–393 days; 9 females), sixteen 18-month-olds (18 M, mean age: 561 days, 549–570 days; 9 females) and sixteen 24 to 25-month-olds (24 M, mean age: 760 days, range: 730–783 days; 11 females). An additional 4 infants were excluded from the final sample because of crying (one 6-month-old, two 18-month-olds and one 24-month-old).

In Experiment 2, participants consisted of 99 healthy, full-term infants divided into three groups based on age: thirty-four 12-month-olds (12 M, mean age: 379 days, range: 365–393 days; 12 females), thirty-three 18-month-olds (18 M, mean age: 563 days, 549–571 days; 15 females) and thirty-two 24 to 25-month-olds (24 M, mean age: 754 days, range: 731–786 days; 19 females). An additional 2 infants were excluded from the final sample because of crying (one 12-month-old and one 24-month-old).

The study was approved by the review board of Osaka University and all experiments were carried out in accordance with the Helsinki Declaration guidelines and regulations. Parents of all participants gave written informed consent before participation.

### Apparatus

The infants sat on their parent’s lap at a distance of 70 cm from a liquid crystal 23-inch monitor (Tobii TX Display, Tobii Technology AB). The spatial resolution of the monitor screen was 1,280 × 720 pixels (39° × 31°). The infant’s eye level was aligned to the centre of the screen at the appropriate height. The parent holding the baby fixated at the centre of the upper side of the monitor throughout the session. The infant’s eye movements were recorded at 120 Hz using an infrared eye tracker (X300, Tobii Technology AB) and a data acquisition software provided by the manufacturer (Tobii Studio, version 3.0). A five-point method implemented in the software was used for calibration. Each experiment was started after the experimenter confirmed that the program collected a sufficient number of stable eye-position data for each eye. To attract infants’ attention, we presented a ball that expanded and contracted with sound as a calibration target. The video stimuli were presented with the Tobii Studio software. The eye movements were analysed offline using Matlab 2015a (Mathworks).

### Stimuli and Procedure

We employed two video stories, one for Experiment 1 and another for Experiment 2, both of which were the same as those used for the previous primate study^13^. Each infant viewed one video story twice, once on Day 1 and once on Day 2 with a 24-hour interval.

**Figure 1:**
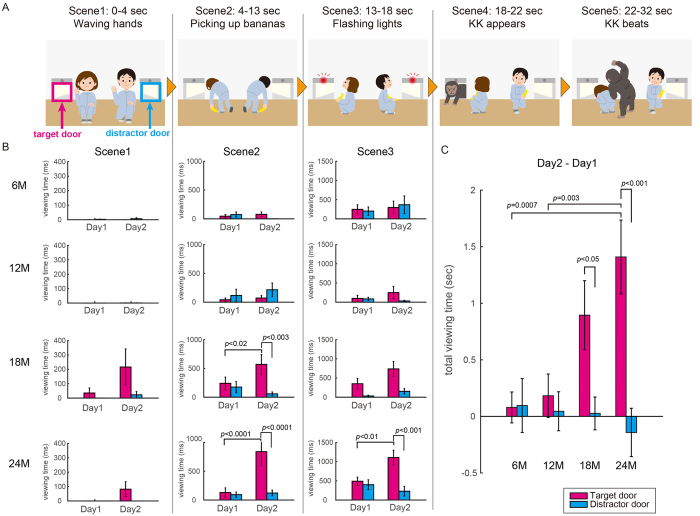
Anticipatory looking behaviour based on place memory. (**A**) Illustrations of a video story used in experiment 1. This video is composed of five scenes: in the first scene, the two human actors said ‘Hi’ by waving their hands (Scene1). In the next scene, they moved to pick up bananas on the floor (Scene2). Then, lights above the left and right doors flashed with alarm sounds (Scene3). Suddenly, King Kong (KK) appeared from one of the two doors and was roaring (Scene4). Finally, KK beat the closest actor and then went back through the same door he came out of (Scene5). Target door refers to the place from which KK appeared (area framed by magenta line) and distractor door refers to the opposite place (area framed by cyan line). The circled areas are the region of interest defined for the doors. Copyrights (c) Akiko Maruo 2016. (**B**) Comparisons of viewing time for each door between the first and second days for each age group in each scene of the video clip. (**C**) Group comparisons of total viewing time, subtracting the first day from the second day for each door. The error bars represent standard errors. P-values represent results of a post-hoc test after the detection of significant interaction in the ANOVA test.

The video story for Experiment 1 (total length: 32 sec) was prepared for testing ‘location’ memory (see Fig. 1A): while two actors sat on the floor, a third actor wearing a costume of King Kong (KK) suddenly appeared from one of two doors and attacked one of the actors on the floor. Then, KK ran away through the door from which he entered. We examined whether the infants would make anticipatory looks at the door on Day 2 before the time of KK’s appearance (0–18 sec, scene1–scene3 in Fig. 1A). We counterbalanced the side of the target door (left or right) across participants, by preparing two versions (left vs. right) of the same story.

**Figure 2:**
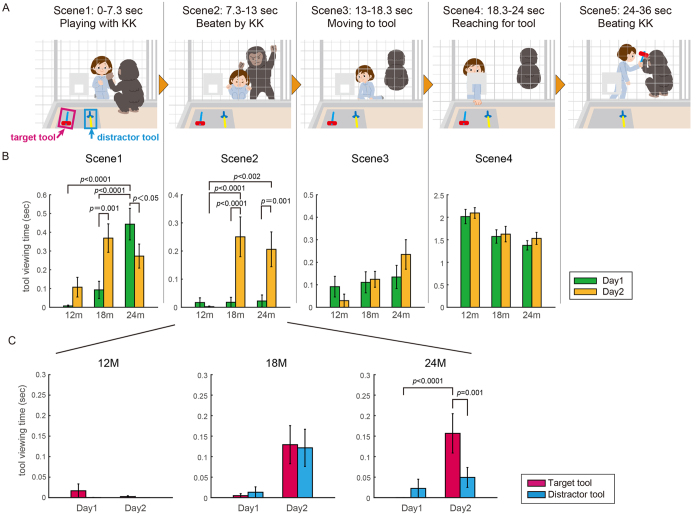
Anticipatory looking behaviour based on object memory. (**A**) Illustrations of a video story used in experiment 2. This movie is composed of five scenes: in the first scene, a female actor played with KK and KK came out from the cage (Scene1). Suddenly, KK beat the actor and then sat down with his back to her (Scene2). Then, the actor slowly moved toward the tools (Scene3) and reached for them (Scene4). After grabbing one of the tools, she hit KK with it and KK ran away (Scene5). The target tool refers to the object which the actor used (framed by the magenta lines) and the distractor tool refers to the opposite object (framed by the cyan lines). The circled areas are the region of interest defined for the tools. Copyrights (c) Akiko Maruo 2016. (**B**) Comparisons of tool viewing time (any tool) between the first and second days across age group for each scene. (**C**) Comparisons of viewing time between the target and the distractor tools across the two days for each age group during Scene2. The error bars represent standard errors. P-values represent results of post-hoc test after a detection of significant interaction in the ANOVA test.

The video story for Experiment 2 (total length: 36 sec) was prepared for testing ‘object’ memory (see Fig. 2A): when a female actor played with KK, KK suddenly started beating the actor with its fists. After KK stopped beating the actor, the actor reached for and grabbed one of two objects (a hammer or a sword made of plastic). Then, the actor hit back at KK using the object. We examined whether the infants, in their second viewing, would make anticipatory looks at the object on Day 2 before the actor grabbed it. To control for the location cue, half of the participants viewed another version of the video in which the location of the two objects (left or right) was switched. The other half of the participants viewed the same video clip on both Day 1 and Day 2. We counterbalanced the target objects (hammer or sword) and the locations of the target object (left or right) across participants, by preparing four (two-by-two) versions of the same story.

### Data analysis

The gaze positions of the right and the left eyes were averaged to yield a single gaze position for each time point. When the gaze position was available from the right or the left eye alone, we used the gaze position of that particular eye. We treated the data as missing, when neither of the data was available. We did not use any temporal interpolations.

In Experiment 1, we defined an area of interest (AOI) for each door (size: 3° × 7°) and analysed the viewing time for each door by counting the number of valid gaze position data each of which represented a frame duration of 1/120 s (8.3 ms). In Experiment 2, we defined AOI for each tool (hammer: 4° × 5°, sword: 4° × 7°) and analysed the viewing time for each tool.

To compare eye-tracking quality across the age groups, we calculated the ratio of valid data points that fell on the monitor screen, which we term the viewing ratio. In Experiment 1, the viewing ratio was greater than 90% in all infants in both Day 1 and Day 2 (Table 1), though the mean ratio was significantly smaller in the youngest group (6 M) than the others (two-way ANOVA (age × day), F_3,60 (age)_ = 5.2, *p* = 0.003; post hoc t-tests, 6M-12M, t_60_ = 2.9, *p* = 0.006; 6M-18M, t_60_ = 2.4, *p* = 0.02; 6M-24M, t_60_ = 3.8, *p* = 0.0004). The mean viewing rate was greater than 90% in any time period of the video clip (0–5 s: 91%, 5–10 s: 97%, 10–15 s: 95%, 15–20 s: 99%, 20–25 s: 99%, 25–30 s: 97%). In Experiment 2, the viewing ratio was greater than 95% in all infants in both Day 1 and Day 2 (Table 1), though the mean ratio was significantly smaller in the youngest group (12 M) than the others (two-way ANOVA (age × day), F_2,96 (age)_ = 6.9, *p* = 0.002; post-hoc t-tests, 12M-18M, t_96_ = 3.3, *p* = 0.002; 12M-24M, t_96_ = 3.1, *p* = 0.003). The mean viewing ratio was greater than 90% in any time period of the video clip (0–5 s: 92%, 5–10 s: 98%, 10–15 s: 98%, 15–20 s: 97%, 20–25 s: 99%, 25–30 s: 99%, 30–35 s: 97%).

We further compared the ratio of the valid face viewing time across the four age groups by defining an AOI on the face of each actor. The proportion of face viewing time to the total length of the video clip (32 sec in Experiment 1) was 0.19 ± 0.07 (mean ± s.d.) in the 6-month, 0.25 ± 0.08 in the 12-month, 0.20 ± 0.05 in the 18-month, and 0.19 ± 0.05 in the 24-month-old infants. Likewise, the proportion of face viewing time in Experiment 2 (36 sec) was 0.27 ± 0.07 in the 12-month, 0.25 ± 0.06 in the 18-month, and 0.24 ± 0.06 in the 24-month-old infants. One-way ANOVAs showed that the proportion of the face viewing time was not significantly different across the four age groups (Experiment 1: F_3,60_ = 1.1, p = 0.37; Experiment 2: F_2,96_ = 0.52, p = 0.60). Considering the fact that the valid data ratio was slightly but significantly smaller in the youngest groups, the lack of significant across-age-group differences in the face viewing time indicates that the youngest infants paid as much attention to the face of each character as the older infants. That is, the youngest groups viewed the video clips in a manner comparable to those in the older infants.

## Results

### Experiment 1

We first examined whether all infants viewed the critical event of the video story on Day 1. Although 36 of 64 infants viewed the opposite side of the target door at the onset of the critical event, all of them immediately shifted their gaze toward the other side where the critical event took place. Thus, we infer that all infants paid attention to the place where the action to-be-remembered took place on Day 1.

Next, to examine whether the infants would make anticipatory looks at the target door on Day 2 before the appearance of KK, we divided the video clips into 5 scenes and analysed the viewing time of each door for each scene (Fig. 1A, Scenes 1–3 before KK’s appearance). As shown in Fig. 1B, the 18- and 24-month-old infants increased their viewing time of the target door on Day 2, especially in Scenes 2 and 3. However, there was little change in their viewing time of the distractor door. In contrast, the 6- and 12-month-old infants did not show such an increase in their viewing time of the target door in these scenes. The two-way analysis of variance (ANOVA) detected a significant interaction between the main factors of the day (Day 1/2) and the door (target/distractor doors) in Scene 2 for the 18-month-old infants (F_1,15_ = 6.0, *p* = 0.03) and in Scenes 2 and 3 for the 24-month-old infants (scene2: F_1,15_ = 10.4, *p* = 0.006; scene3: F_1,15_ = 5.1, *p* = 0.04). Post-hoc tests confirmed significant increases in the viewing time of the target door on Day 2 as compared to the viewing time of the distractor door on Day 2 (18 M, scene2: F_1,30_ = 10.7, *p = *0.0028; 24 M, scene2: F_1,30_ = 13.7, *p = *0.0009; 24 M, scene3: F_1,30_ = 15.4, *p = *0.0005) and as compared to the viewing time of the target door on Day 1 (18 M, scene2: F_1,30_ = 6.1, *p < *0.02; 24 M, scene2: F_1,30_ = 26.9, *p < *0.0001; 24 M, scene3: F_1,30_ = 8.3, *p = *0.007). The viewing time of the target door on Day 2 gradually increased during the first three scenes in both the 18- and 24-month-old infants (see Supplementary Figs 1 and 2). Thus, the increase in the total viewing time of the target door from Day 1 to Day 2 was markedly greater than that of the distractor door in the 18- and 24-month-old groups (Fig. 1C). In contrast, the infants in the 6- and 12-month-old groups showed little change in the total viewing time across Day 1 and Day 2 for either of the two doors. The two-way ANOVAs (age group x doors) revealed a significant interaction between the main factor of the age group (6-, 12-, 18- and 24-month-old) and the main factor of the door (F_3, 60_ = 3.4, *p = *0.037). Post-hoc tests revealed that the across-day increase in the viewing time for the target door was significantly greater than that for the distractor door in the 18-month-old (F_3, 60_ = 5.9, *p = *0.018) and 24-month-old infants (F_3, 60_ = 13.6, *p* = 0.0005). In addition, the across-day increase for the target door in the 24-month-old infants was significantly greater than that in the 6-month-old (t_30_ = 3.8, *p = *0.0007, t-test) and 12-month-old infants (t_30_ = 3.2, *p = *0.003, t-test).

We also conducted a three-way ANOVAs with factors of age, day and door for each scene. As a result, a significant interaction of age with day and door was observed in Scene 2 of Experiment 1 (F_3,60_ = 3.9, p = 0.01) but not in Scene 1 (F_3,60_ = 2.4, p = 0.08) and Scene 3 (F_3,60_ = 1.3, p = 0.3). Post-hoc test revealed that a significant age difference was observed in the viewing time of the target door on Day 2 (F_3,240_ = 15.2, p = 0.001).

### Experiment 2

We first confirmed that all infants viewed the critical action of the actor on Day 1 when the actor reached to and grasped one of the tools. Then, we divided the video clips into 5 scenes and examined whether the infants would increase their viewing time of the tools as a whole (without discrimination between the hammer and the sword) before the actor picked it up on Day 2 (Fig. 2A: scenes1–4). As shown in Fig. 2B (and see also Supplementary Figs 3 and 4), the tool viewing time was remarkably increased from Day 1 to Day 2 during the first two scenes in the 18-month-olds. The 24-month-old infants also increased their tool viewing time on Day 2 during Scene 2, though their tool viewing time on Day 2 decreased during Scene 1. By contrast, the 12-month-old infants did not show any significant changes in either scene. The two-way ANOVA detected a significant interaction between the age and the day in Scene 1 (F_2, 96_ = 7.4, *p* = 0.0011) and Scene 2 (F_2, 96_ = 5.8, *p* = 0.0041). Post-hoc tests of Scene 1 confirmed that the tool viewing time was significantly increased from Day 1 to Day 2 in the 18-month-old infants (F_1, 96_ = 11.1, *p* = 0.0012). By contrast, the tool viewing time dropped significantly from Day 1 to Day 2 in the 24-month-old infants (F_1, 96_ = 4.2, *p* = 0.043). Their tool viewing time on Day 1 was significantly greater than that of the 12-month-old (t_64_ = 5.1, *p* < 0.0001, *t-test*) and 18-month-old infants (t_63_ = 3.5, *p* < 0.0001, *t-test*). That the 24-month-old infants spent more time on viewing tools on Day 1 as compared to younger children suggests that general interests in tools increase during the second year of life. However, it is also worth noting that the effect of general interests shown by the 24-month-olds disappeared on Day 2 as long as Scene 1 is concerned. On the other hand, post-hoc tests of Scene 2 confirmed that the increase in the tool viewing time from Day 1 to Day 2 was significant in both the 18-month-old (F_1, 96_ = 18.4, *p* < 0.0001) and 24-month-old infants (F_1, 96_ = 11.5, *p* = 0.001). In addition, the tool viewing time on Day 2 was significantly greater in both the 18- and 24-month-old infants as compared to the 12-month-old infants (18 M: t_65_ = 3.5, *p* < 0.0001; 24 M: t_64_ = 3.2, *p* = 0.0019, *t-test*).

Next, we examined whether the infants would increase their viewing time of the target tool in particular. During Scene 2, when both the 18- and 24-month-olds showed significant increases in the tool viewing time as a whole, only the 24-month-old infants increased their viewing time of the target tool in particular (Fig. 2C, 24 M). Although the 18-month-old infants increased their tool viewing time as a whole, they showed no preference for the target tool (Fig. 2C, 18 M). The two-way ANOVA detected a significant interaction between the day and the tool only during Scene 2 in the 24-month-old infants (F_1, 62_ = 6.0, *p* = 0.02), but not in any other combination of the scenes and participant groups (see Supplementary Fig. 5). In the post-hoc test of Scene 2 in the 24-month-old infants, the viewing time of the target tool on Day 2 was significantly greater than that of the distractor tool on Day 2 (F_1, 62_ = 11.2, *p* = 0.0014) and greater than that of the target tool on Day 1 (F_1, 62_ = 14.2, *p* = 0.0004). In this experiment, to examine the effect of location memory on object memory, we presented to half of the infants the video in which the locations of the tools were switched on Day 2. However, we did not observe any effect of location switch on the tool viewing time in this scene using three-way ANOVAs with factors of switch (same/switch), day (Day 1/Day 2) and tool (target/distractor). This suggests that the 24-month-old infants showed a preference for the target tool independent of its location memory. Finally, we also conducted a three-way ANOVAs with factors of age, day and tool. However no significant interactions of age with day or with tool was observed (F_2,93_ = 2.4, *p* = 0.1).

## Discussion

The developmental process of episodic-like memory in preverbal infants under 2 years old remains unknown. To address this issue, the present study examined the anticipatory looking behaviour of preverbal infants toward events that they encountered only once, 24 hours earlier. In the first experiment, the 18 and 24 months old infants looked at the location where the character would show up in their second viewing before the critical event onset. However, the 6 and 12 months old infants did not show such behaviour. This result suggests that infants made anticipatory looks toward the target location based on long-term memory for *where* information by 18 months after birth. In the second experiment, when the human actor was beaten by the animal character on the second day, the infants older than 18 months increased their viewing time on the tools before the actor actually held one of the tools. This result suggests that the attack from the animal character prompted infants to recall a memory that the human actor had used a tool for revenge and thus to make anticipatory looks toward the tools. Moreover, the infants over 24 months old looked at the tool that the human actor had used on Day 1, though those who were 18 months old did not show any preference for a particular tool. This result implies that the 24-month-old infants formed a more precise memory for object information than did the 18-month-old infants. Taken together, the present results demonstrate that infants develop an ability to encode and retrieve the where and what of an event that they experienced only once, which is, long-term memory of a one-time event, at the age of 18 months and elaborate this ability over the next 6 months.

One might argue that a technique using deferred imitation has already demonstrated the existence of event-based long-term memory in much younger infants. Deferred imitation examines whether infants reproduce previously observed actions to objects at a later time. Previous studies reported that infants as young as 6 and 9 months of age exhibited deferred imitation of actions with objects after an interval of 24 hours^10^,^19^. Because deferred imitation is based on an observation of the target actions without training and depends on the context, many researchers argued that this deferred imitation taps the ability of declarative memory but not procedural memory in preverbal infants^5^,^19^. However, in contrast to the early emergence of long-term memory retention in the deferred imitation studies, the present study revealed that the infants under 12 months did not make anticipatory looks based on the long-term memory of a previous single experience. The most important factor that caused the difference would be the number of repetition: the same action was presented several times in the deferred imitation studies^5^,^10^,^20^, but only once in the present study. In fact, the number of repetition was critical for 6-month-old infants in particular^10^. They failed to show deferred imitation when an action was demonstrated three times, but showed imitation only when the number of repetition was increased to six. It is also worth noting that there is no previous evidence that even 18-month-olds could show deferred imitation when an action was presented only once. We have here shown that the 18-month-old infants are able to show the anticipatory looking behaviour to a particular position (Experiment 1) or to objects related to a future event (Experiment 2) at a particular timing (both experiments), even when the event was presented only once. Thus the present study has provided compelling evidence that children at the age of 18 months has a kind of episodic-like memory, which is an ability to integrate the what, where and when aspects of a unique past event^7^,^21^.

Several factors other than the number of repetition, should also be considered. First, the action was presented live in the deferred imitation studies, but on screen in the present study. Demonstration of an action in the live format has been shown to be more effective to infants for inducing imitation of the action as compared to demonstration on screen^22^,^23^. Second, there was little emotional factor in the demonstration of action in the deferred imitation studies, but there was some in the present study. It is generally accepted that emotional contexts enhance long-term memory even when the context is consciously recognized or not^16^. This is true for preverbal infants: four-month-old infants were reported to show autonomic or cortisol responses when they were placed in a socially stressful context that they experienced two weeks before^17^,^18^. Since the emotional content is potentially better understood and hence more salient to the older children, there is a possibility that infants younger than 18 months old would have shown anticipatory looks, had a better story been presented in a live format even if it was presented only once. Optimal conditions for the content and format of presentation do merit further investigation.

Previous studies using the same stimuli and paradigm for great apes reported that they showed anticipatory looking behaviour just before the event onset: when the red light above the door was alarming or when the human actor reached for the objects^13^. These percussive events provided cues for recalling the impending event memory in the great apes. In contrast, preverbal infants in the present study showed anticipatory looking behaviour earlier in time before the salient cues were presented. This difference suggests that human infants recall past events by contextual information but that great apes depend on the associative memory between the cue and event. Despite the same anticipatory looks based on the long-term event memory, there is a possibility that systems of episodic-like memory are somewhat different between non-verbal animals and preverbal human infants.

In summary, our study has shown that preverbal infants over 18 months of age encode the information of a one-time event in a movie and retrieve it to guide anticipatory eye-movements for future events. The present study demonstrated that this paradigm contributes to the understanding of event-based long-term memory regardless of language ability not only for non-verbal animals but also for preverbal infants. The eye-movement behaviour affected by memory occurred even in the absence of awareness^24^. This suggests that this paradigm can be used as a more veridical and robust index of long-term event memory than behavioural reports alone even for human adults, especially those with amnesia, as well as for preverbal infants.

## Additional Information

**How to cite this article**: Nakano, T. and Kitazawa, S. Development of long-term event memory in preverbal infants: an eye-tracking study. *Sci. Rep.* **7**, 44086; doi: 10.1038/srep44086 (2017).

**Publisher's note:** Springer Nature remains neutral with regard to jurisdictional claims in published maps and institutional affiliations.

## Supplementary Material

Supplementary Figures

## Figures and Tables

**Table 1:** Comparison of the viewing ratio (%) across the age groups (mean ± s.d.).

	Experiment 1		Experiment 2	
	Day 1	Day 2	Day 1	Day 2
6 M	92 ± 6	94 ± 6	—	—
12 M	98 ± 1	97 ± 4	95 ± 7	95 ± 10
18 M	95 ± 10	98 ± 1	98 ± 3	98 ± 1
24 M	98 ± 2	99 ± 2	98 ± 3	98 ± 3
