# Supplementary material for: Development of long-term event memory in preverbal infants: an eye-tracking study
Source: Sci Rep. 2017 Mar 8;7:44086. doi: 10.1038/srep44086 (PMC5341052; doi:10.1038/srep44086)

Development of long-term event memory in preverbal infants: an eye-tracking study

Tamami Nakano, Shigeru Kitazawa

Supplementary Figure 1. **Viewing rate along the timeline of the video clip before the critical event onset in experiment 1**. The magenta and the cyan lines represent the viewing rate of the target and the distractor doors, respectively (number of infants viewing the door/number of infants). The x-axis represents the timeline of the video clip (Scene1–Scene3). The panels at the top and in the second rows show the results of the first day and the second day for each age group, respectively. The panels in the bottom row show the viewing rate, subtracting the first day from the second day.

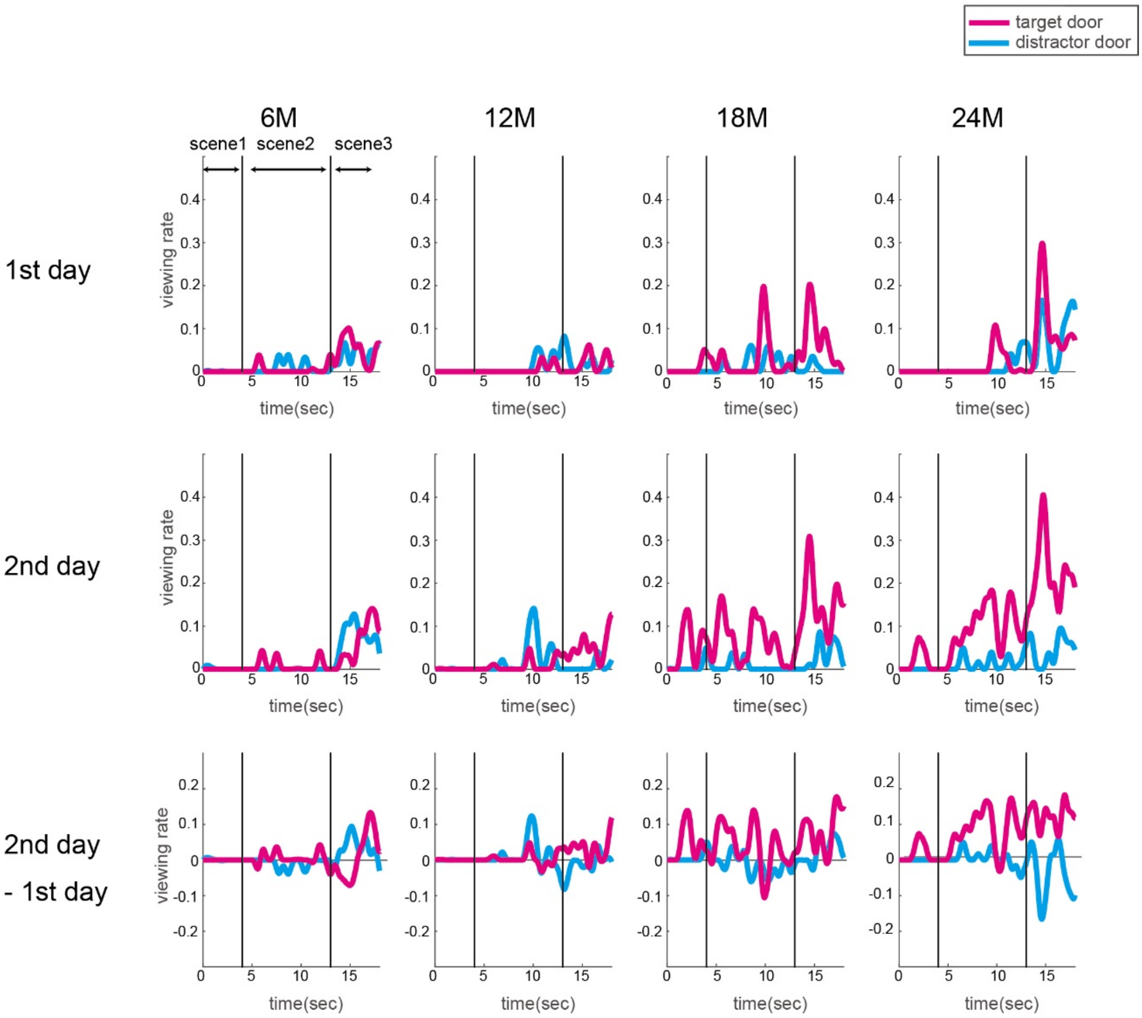
target door
distractor door
6M
12M
18M
24M
scene1
scene2
scene3
1st day
2nd day
2nd day
- 1st day
viewing rate
time(sec)

Supplementary Figure 2. **Accumulation of viewing time along the timeline of the video clip before the critical event onset in experiment 1**. The magenta and the cyan lines represent the accumulations of viewing time for the target and the distractor doors, respectively. The x-axis represents the timeline of the video clip (Scene1–Scene3). The panels at the top and in the second rows show the results of the first day and the second day for each age group, respectively. The panels in the bottom row show the viewing rate, subtracting the first day from the second day.

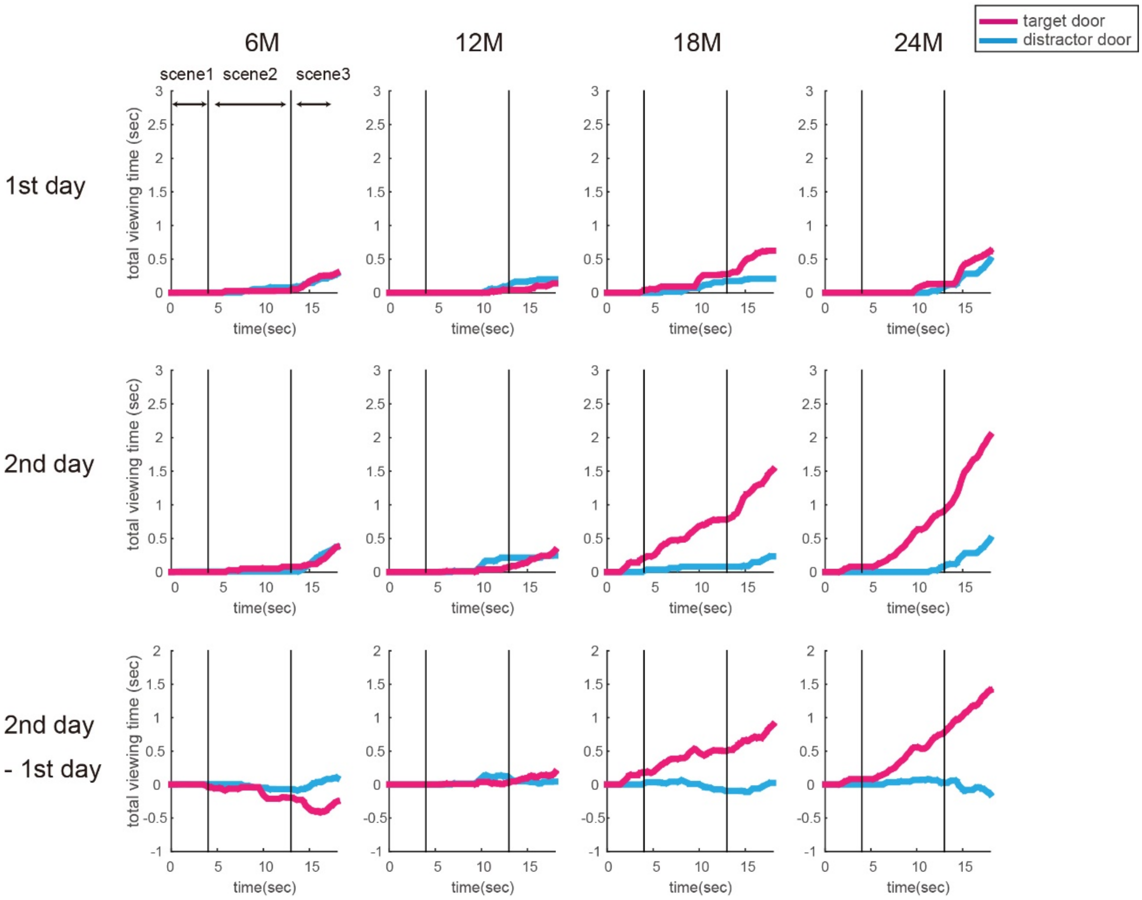

Supplementary Figure 3. **Viewing rate along the timeline of the video clip before the critical event onset in experiment 2**. The magenta and the cyan lines represent the viewing rate of the target and the distractor tools, respectively (number of infants viewing the tool/number of infants). The x-axis represents the timeline of the video clip (Scene1–Scene4).The panels at the top and in the second rows show the results of the first day and the second day for each age group, respectively. The panels in the bottom row show the viewing rate, subtracting the first day from the second day.

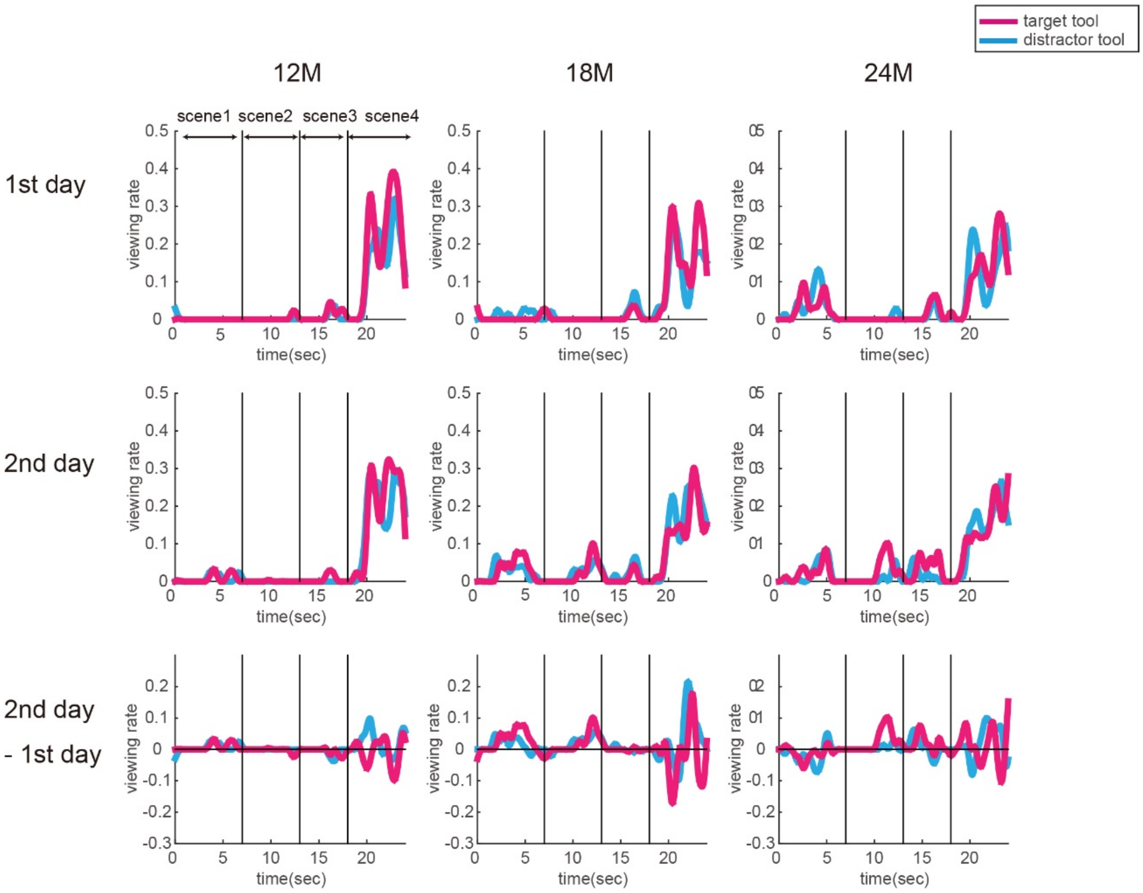

Supplementary Figure 4. **Accumulation of viewing time along the timeline of the video clip before the critical event onset in experiment 2**. The magenta and the cyan lines represent the accumulations of viewing time for the target and the distractor doors, respectively. The x-axis represents the timeline of the video clip (Scene1–Scene4).The panels at the top and in the second rows show the results of the first day and the second day for each age group, respectively. The panels in the bottom row show the viewing rate, subtracting the first day from the second day.

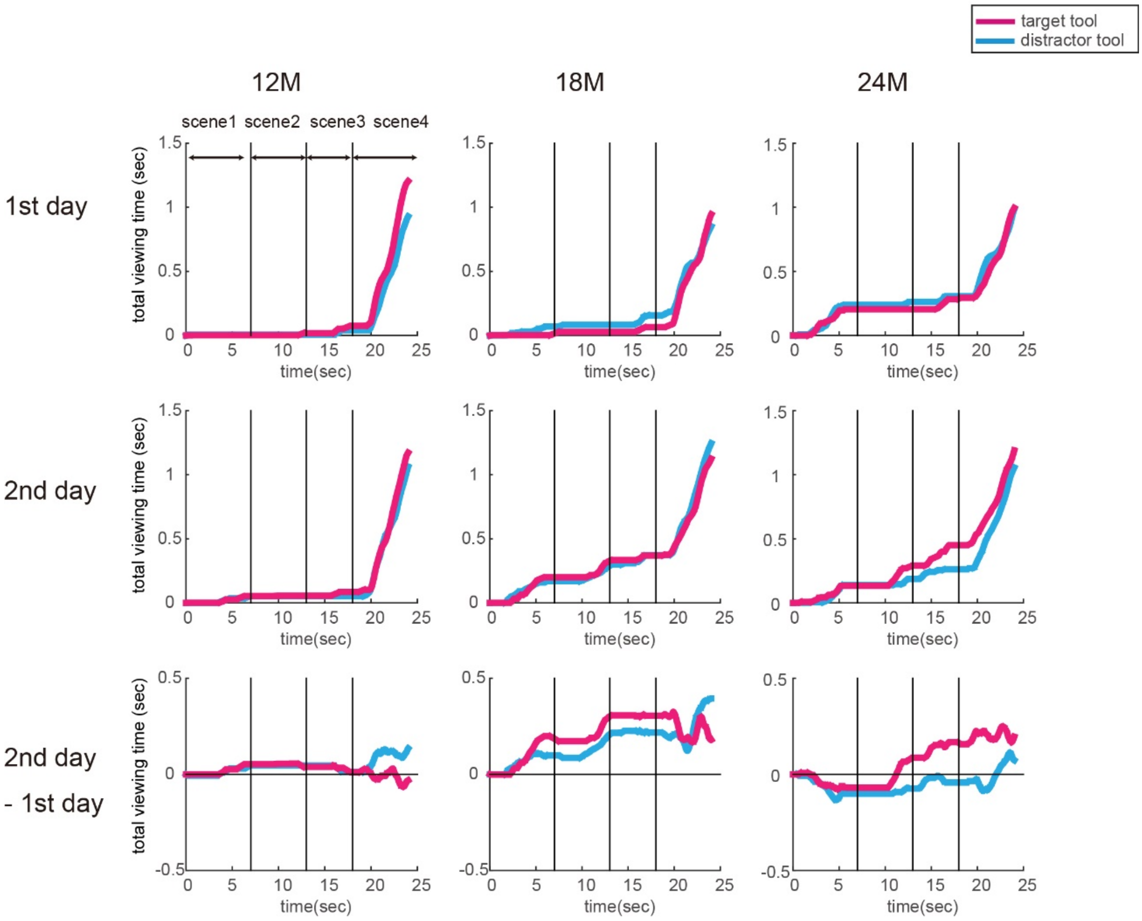

Supplementary Figure 5. **Comparisons of viewing time between the target and the distractor tools for each scene in each age group in experiment 2.** The error bars represent standard errors.

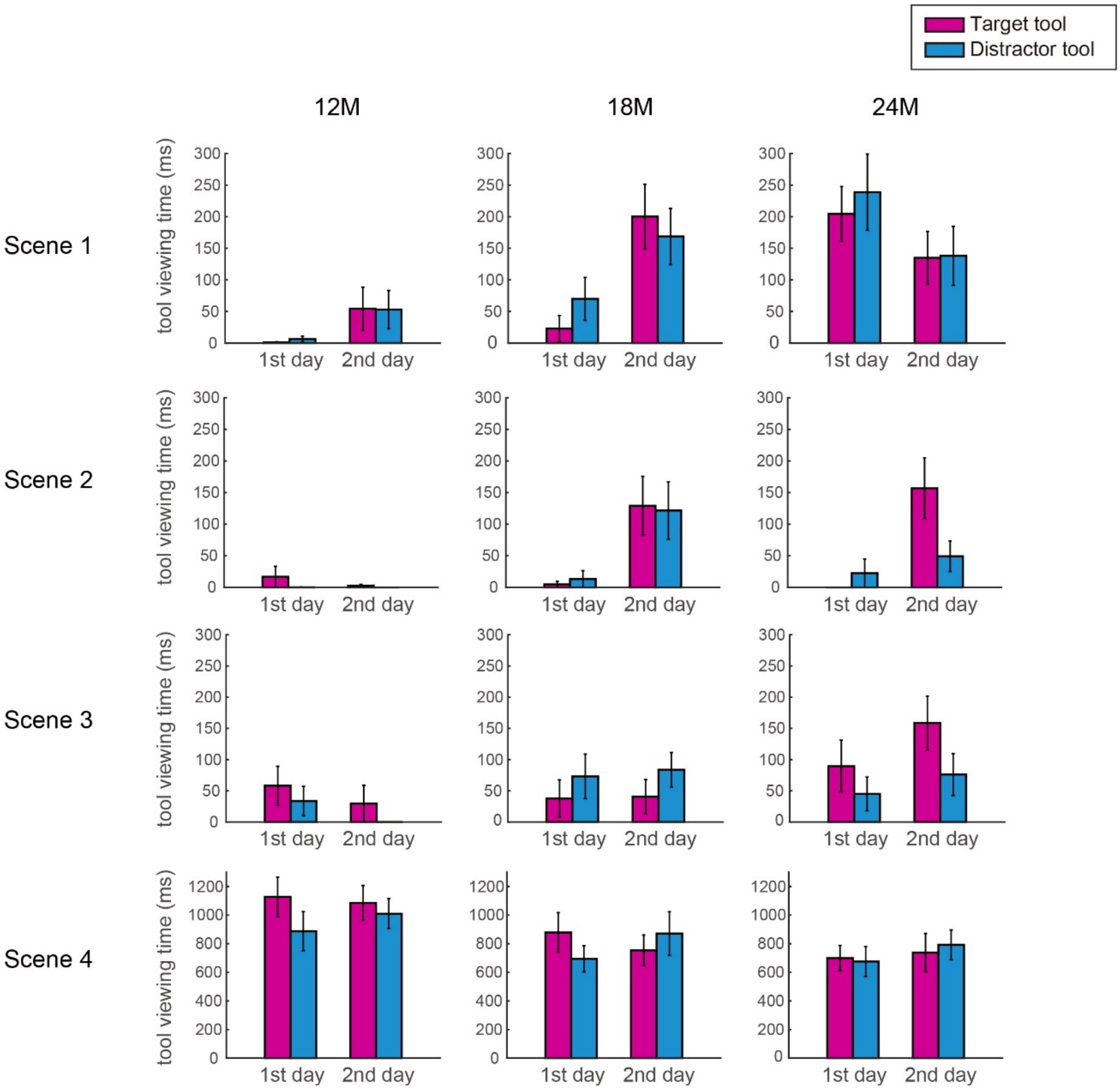

Supplement: Supplementary Figures [file srep44086-s1.pdf]
